# On the transformation of MinHash-based uncorrected distances into proper evolutionary distances for phylogenetic inference

**DOI:** 10.12688/f1000research.26930.1

**Published:** 2020-11-10

**Authors:** Alexis Criscuolo

**Affiliations:** 1 Hub de Bioinformatique et Biostatistique - Département Biologie Computationnelle, Institut Pasteur, USR 3756, CNRS, 75015 Paris, France

**Keywords:** MinHash, p-distance, evolutionary distance, substitution model, phylogenetics, genome, simulation

## Abstract

Recently developed MinHash-based techniques were proven successful in quickly estimating the level of similarity between large nucleotide sequences. This article discusses their usage and limitations in practice to approximating uncorrected distances between genomes, and transforming these pairwise dissimilarities into proper evolutionary distances. It is notably shown that complex distance measures can be easily approximated using simple transformation formulae based on few parameters. MinHash-based techniques can therefore be very useful for implementing fast yet accurate alignment-free phylogenetic reconstruction procedures from large sets of genomes. This last point of view is assessed with a simulation study using a dedicated bioinformatics tool.

## Introduction

To estimate the level of proximity between two non-aligned genome sequences *x* and *y*, recent methods (e.g. 1– 7) have focused on decomposing the two genomes into their respective sets *K _x_* and *K _y_* of non-duplicated nucleotide *k*-mers (i.e. oligonucleotides of size *k*). A pairwise similarity may then be easily estimated based on the Jaccard index *j* = | *K _x_* ∩ *K _y_*| */ *| *K _x_* ∪ *K _y_*| ^8^. The Jaccard index between two sets of *k*-mers is a useful measure for two main reasons. First, it can be quickly approximated using MinHash-based techniques (MH ^9^), as implemented in e.g. Mash ^2^, sourmash ^3^, Dashing ^4^, Kmer-db ^6^, FastANI ^5^, or BinDash ^7^. Such techniques select a small subset (of size *σ*) of hashed and sorted *k*-mers (called sketch) from each *K _x_* and *K _y_*, and approximate *j* by comparing these two subsets (for more details, see 2, 9– 12). Second, the proportion *p* of observed differences between the two aligned genomes (often called uncorrected distance or *p*-distance) can be approximated from *j* (therefore without alignment) with the following formula (e.g. 13, 14):

$$p=1-{\left(\frac{2j}{j+1}\right)}^{1/k},\;(1)$$

provided that both sizes *σ* and *k* are large enough, and *j* is not too low (see below).

As a consequence, a pairwise evolutionary distance *d* can be derived from the Jaccard index *j* using transformation formulae of the following form:

$$d=-b_{1}\;\log_{e}(1-p/b_{2}),\;(2)$$

where *p* is obtained using Equation (1). Parameters *b*_1 _and *b*_2 _can be defined according to explicit models to estimate the number *d* of nucleotide substitutions per character that have occurred during the evolution of the sequences *x* and *y*, e.g. 15– 24. When *b*_1 _= *b*_2 _= 1, Equation (2) corresponds to the Poisson correction (PC; e.g. 21) distance. Although it is based on a simplistic model of nucleotide substitution ^1, 16, 25, 26^, PC is the *p*-distance transformation implemented in many MH tools (e.g. Mash, Dashing, FastANI, Kmer-db, BinDash). However, more accurate distance estimates may be obtained by using substitution models based on more parameters. Among these models, equal-input (EI, sometimes called F81 ^18, 19, 24, 27– 29^) takes into account the equilibrium frequency *π*_r _of each residue r in Σ = { A, C, G, T}. An EI distance can be estimated using Equation (2) with $b_{1}=1-\sum_{\text{r}\in \Sigma }\pi_{\text{r}}^{2}$ and $b_{2}=1-\sum_{\text{r}\in \text{Σ}}\pi_{{\text{r}}_{x}}\pi_{{\text{r}}_{y}},$ where $\pi_{{\text{r}}_{x}}$ and $\pi_{{\text{r}}_{y}}$ are the frequencies of r in the two sequences *x* and *y*, respectively ^20^. Further assuming that the heterogeneous replacement rates among nucleotide pairs and sites can be modelled with a Γ distribution, an EI distance *d* can be derived from *p* using the following formula:

$$d=ab_{1}[{\left(1-p/b_{2}\right)}^{-1/a}-1],\;(3)$$

where *a >* 0 is the (unknown) shape parameter of the Γ distribution, e.g. 22, 24, 30– 33. It is worth noticing that when *a* is high, Equation (2) and Equation (3) yield very similar distance estimates (for any fixed *b*_1 _and *b*_2_).

The aim of this study is to assess the accuracy of Equation (2) and Equation (3) in transforming a MH *p*-distance $\hat{p}$, where $\hat{p}$ is derived from the MH Jaccard index $\hat{j}$ using Equation (1). In the following, analyses of large sets of simulated nucleotide sequences show three complementary results. First, current MH implementations enable *p*-distances to be conveniently estimated under several conditions. Second, PC and EI transformations (2) and (3) of MH *p*-distance estimates can suitably approximate evolutionary distances derived from general time reversible (GTR; e.g. 34) models of nucleotide substitution. Third, PC and EI distances derived from MH estimates enable accurate phylogenetic tree reconstruction from unaligned nucleotide sequences.

## Results and discussion

### MinHash-based *p*-distance approximation

Varying *d* from 0.05 to 1.00 (step = 0.05), a total of 200 nucleotide sequence pairs with *d* substitution events per character were simulated under the models GTR and GTR+Γ. Each model was adjusted with three different equilibrium frequencies: equal frequencies ( *f*_1_; *π*_A _= *π*_C _= *π*_G _= *π*_T _= 25%), GC-rich ( *f*_2_; *π*_A _= 10%, *π*_C _= 30%, *π*_G _= 40%, *π*_T _= 20%), and AT-rich ( *f*_3_; *π*_A _= *π*_T _= 40%, *π*_C _= *π*_G _= 10%). The GTR substitution rates and the Γ shape parameters were obtained based on a maximum likelihood (ML) analysis of 142 real-case phylogenomics datasets. Overall, ML estimates of Γ shape parameters were quite low (i.e. varying from 0.162 to 0.422, with an average of 0.314), confirming that the heterogeneity of the substitution rates across sites is a non-negligible factor when studying evolutionary processes. Every simulation was completed with indel events, resulting in sequences > 3 Mbs with relative lengths (i.e. longer/shorter) varying from 1.0196 ( *d* = 0.05) to 1.1117 ( *d* = 1.00), on average.

For each of the 2 (GTR, GTR+Γ) × 3 ( *f*_1_, *f*_2_, *f*_3_) × 20 ( *d* = 0.05, 0.10, ..., 1.00) × 200 = 24,000 simulated sequence pairs *x* and *y*, the corresponding *p*-distance was estimated using three MH tools: Mash, BinDash and Dashing. Of note, the accuracy of a MH estimate $\hat{j}$ of the Jaccard index between *K _x_* and *K _y_* is mainly dependent on two parameters: the *k*-mer size *k* and the sketch size *σ*. The size *k* should be large enough to minimize the probability *q* of observing a random *k*-mer shared by *x* and *y* by chance alone. Such a value can be obtained from *q* by *k* = ⌈log _|Σ| _ ( *g*(1− *q*) */q*) − 0.5⌉, where *g* is the length of the largest sequence ^2, 29, 35^. The size *σ* should be large enough to minimize the error bounds of $\hat{j}$ ^2^, but also to avoid the inconvenient estimate $\hat{j}$ = 0. Following 29, *σ* was set by the proportion *s* of the average sequence length.

To investigate the impact of both parameters *σ* and *k* on the accuracy of the MH estimates, each MH tool was used with *s* = 0.2, 0.4, 0.6, 0.8 and *q* = 10 ^−3^, 10 ^−6^, 10 ^−9^, 10 ^−12^. As in simulated sequences, *g* ranges from 4.99 Mbs ( *d* = 0.05) to 3.38 Mbs ( *d* = 1.00) on average, *s* translates into moderately to very large sketch sizes *σ*, and *q* into *k*-mer sizes *k* = 16, 21, 26, 31.

**Figure 1.**
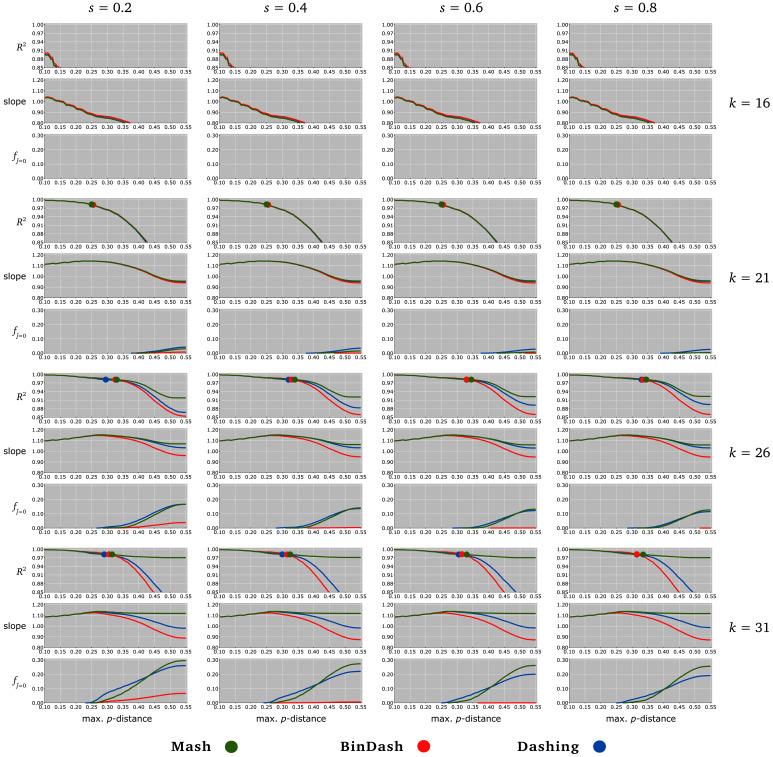
Accuracy of MH tools for estimating *p*-distances from unaligned nucleotide sequences. For each sketch size (columns; set by *s* = 0.2, 0.4, 0.6, 0.8) and each *k*-mer size (rows; *k* = 16, 21, 26, 31), three line charts represent different statistics determined with Mash (green), BinDash (red), and Dashing (blue). For *p*_max _ranging from 0.10 to 0.55 ( *x*-axes), represented statistics are (i) the coefficient of determination *R* ^2 ^(up; *y*-axis ranging from 0.85 to 1.00) and (ii) the slope of the linear least-square regression through the origin (middle; *y*-axis ranging from 0.8 to 1.2) computed from estimated $\hat{p}$ and corresponding ’true’ *p*-distances *p* ≤ *p*_max_, as well as (iii) the cumulative proportion $f_{\hat{j}=0}$ of estimated Jaccard index $\hat{j}$ = 0 within [0, *p*_max_] (bottom; *y*-axis ranging from 0.0 to 0.3). Circles in *R* ^2 ^line charts (up) indicate the largest value *p _r>_*_0.99 _such that the subset of pairs ( *p*, $\hat{p}$) defined by *p* ≤ *p _r>_*_0.99 _provides a coefficient of correlation *r >* 0.99.

**Figure 2.**
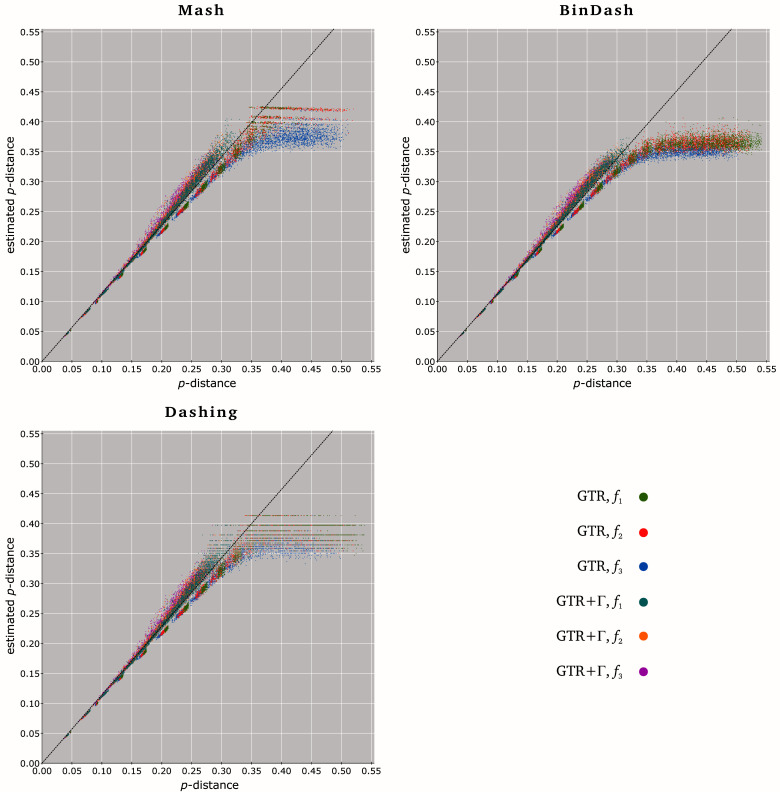
MH *p*-distance estimates between 24,000 pairs of unaligned nucleotide sequences. The *p*-distances $\hat{p}$ estimated by Mash (up left), BinDash (up right) and Dashing (bottom left) with *k* = 26 ( *q* = 10 ^−9^) and *s* = 0.8 are plotted against the ’true’ *p*-distances *p* between 24,000 pairs of nucleotide sequences simulated under six scenaria of evolution: GTR with equilibrium frequencies *f*_1 _= (0.25, 0.25, 0.25, 0.25) (green points), *f*_2 _= (0.10, 0.30, 0.40, 0.20) (red) and *f*_3 _= (0.40, 0.10, 0.10, 0.40) (blue), and GTR+Γ with *f*_1 _(cyan), *f*_2 _(orange) and *f*_3 _(magenta). Points corresponding to $\hat{j}$ = 0 are not represented. Each scatter plot is completed with the least-square regression line through the origin (dashed black line) estimated from the subset of points ( *p*, $\hat{p}$) such that *p* ≤ *p _r>_*_0.99_, where *p _r>_*_0.99 _= 0.345 (Mash), 0.335 (BinDash) and 0.330 (Dashing).

Two statistics were calculated to assess the linear relationship between the MH estimate $\hat{p}$ (derived from $\hat{j}$≠ 0) and the ’true’ *p*-distance *p*: the coefficient of determination *R* ^2 ^and the slope *β* of the linear least-square regression $\hat{p}$ = *βp*. Let Ψ( *p*_max_) be the subset of pairs ( *p*, $\hat{p}$) such that *p* ≤ *p*_max_. Varying *p*_max _from 0.10 to 0.55, *R* ^2^ and *β* were estimated from Ψ( *p*_max_) ( Figure 1). The cumulative proportions $f_{\hat{j}=0}$ of MH Jaccard index $\hat{j}$ = 0 within [0, *p*_max_] were also measured ( Figure 1). Finally, every value *p _r>_*_0.99 _was estimated, where *p _r>_*_0.99 _is defined as the highest *p*-distance such that the subset Ψ( *p _r>_*_0.99_) provides a coefficient of correlation *r >* 0.99 (as assessed by a Fisher transformation *z*-test with *p*-value < 1%; Figure 1). The highest values *p _r>_*_0.99 _were obtained with parameters *k* = 26 ( *q* ≤ 10 ^−9^) and *s* = 0.8 (illustrated in Figure 2).

One important result ( Figure 1) is that current MH implementations return suitable estimates of *p* as long as *p* ≤ 0.25, provided that *k* is sufficiently large. Indeed, when *k* ≥ 21 (and any *s* ≥ 0.2), the statistics *p _r>_*_0.99 _are higher than 0.25 ( Figure 1), therefore showing that *p* and $\hat{p}$ are highly linearly correlated when *p* ≤ 0.25 (see e.g. Figure 2). Interestingly, when *p* ≤ 0.25, the worthless estimate $\hat{j}$ = 0 was almost never observed with the different selected parameters *s* and *q* ( Figure 1).

Furthermore, when *p >* 0.25, large *k*-mers are required to obtain satisfactory estimates, i.e. *k >* 21 or *q <* 10 ^−6 ^( Figure 1). However, dealing with *k >* 21 involves using large sketch sizes to minimize the cases $\hat{j}$ = 0 (see $f_{\hat{j}=0}$ in Figure 1). Simulation results suggest that *k* = 26 (i.e. *q* = 10 ^−9^) and *s >* 0.4 yield suitable estimates of *p*, obtained from sequences of lengths > 4 Mbs with pairwise *p <* 0.35 (see Figure 1 and Figure 2). Indeed, when *p* ranges between 0.25 and 0.35, small sizes *k* (e.g. *k* ≤ 21 or *q* ≥ 10 ^−6^) always provide underestimated $\hat{p}$ (with any *s*). Large size *k* (e.g. *k* = 31 or *q* = 10 ^−12^) results in the same trend, but also in high numbers of useless estimates $\hat{j}$ = 0 (even with large *σ*; see $f_{\hat{j}=0}$ in Figure 1).

When *p* ≥ 0.35, MH tools always underestimate the *p*-distances between the sequences simulated for this study ( Figure 1 and Figure 2). One could suggest that more accurate MH estimates $\hat{p}$ will be expected with larger sketch sizes *σ*. Nevertheless, results represented in Figure 2 (i.e. *q* = 10 ^−9 ^and *s* = 0.8, providing the highest *p _r>_*_0.99_) are based on average *σ* varying from ∼2.7 × 10 ^6 ^( *d* = 1.00) to ∼3.6 × 10 ^6 ^( *d* = 0.35), which are larger than some real genomes.

### Transformation of *p*-distances into evolutionary distances

**Figure 3.**
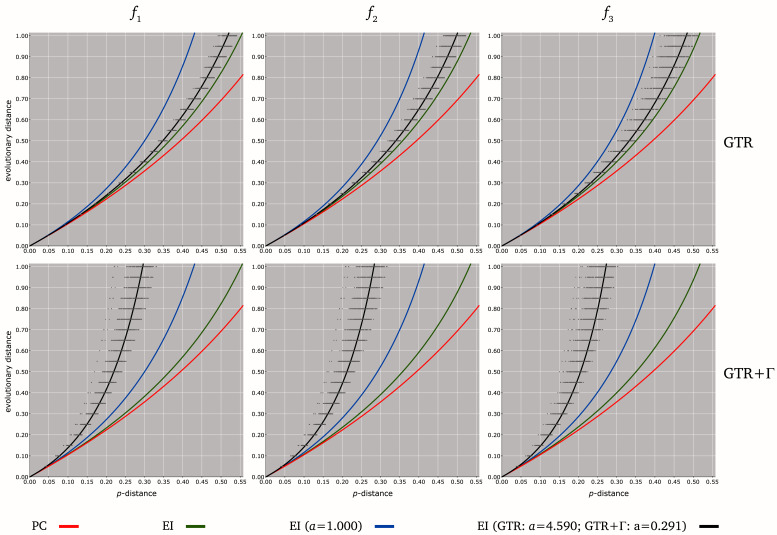
Relationships between *p*-distances and different evolutionary distance estimates under various models of nucleotide substitution. The six charts represent the evolutionary distance *d*( *y*-axis ranging from 0.00 to 1.00) against the *p*-distance *p* ( *x*-axis ranging from 0 to 0.55). Gray points ( *p*, *d*) are derived from the simulation of sets of 4,000 sequence pairs, each under six different scenaria of evolution: GTR (top) and GTR+Γ (bottom) with equilibrium frequencies *f*_1 _= (0.25, 0.25, 0.25, 0.25) (left), *f*_2 _= (0.10, 0.30, 0.40, 0.20) (middle) and *f*_3 _= (0.40, 0.10, 0.10, 0.40) (right). PC and EI versions of Equation (2) are represented with red and green curves, respectively. EI version of Equation (3) is represented with blue curves for *a* = 1, and with black curves for the values *a* = 4.590 and *a* = 0.291 determined by least-square regression from the gray points derived from the models GTR (top) and GTR+Γ (bottom), respectively.

When a pairwise *p*-distance *p* can be estimated from unaligned nucleotide sequences, it may be transformed into an evolutionary distance *d*, based on Equation (2) or Equation (3). The relationship between *p* and *d* was represented in Figure 3 for different distance estimators: PC transformation (2) ( *b*_1 _= *b*_2 _= 1), and EI transformations (2) and (3) with equilibrium frequencies *f*_1 _( *b*_1 _= *b*_2 _= 0.75 under homogeneous substitution pattern ^20^), *f*_2_ ( *b*_1 _= *b*_2 _= 0.70) and *f*_3_ ( *b*_1 _= *b*_2 _= 0.66). Parameter *a* in EI Equation (3) was estimated by least-square regression from the pairs ( *p*, *d*) derived from the sequences simulated under the models GTR and GTR+Γ (see above).

PC and EI *p*-distance transformations (2) result in improper underestimates as the expected distance *d* increases. Indeed, when compared with realistic GTR-based distances *d*, PC and EI transformations (2) give distance estimates that are always lower than *d*, especially under GTR+Γ and when *d* is large (e.g. *d >* 0.1; Figure 3). This downward bias is somewhat expected, knowing that PC and EI transformations (2) are based on less parameters than both models GTR and GTR+Γ. However, the additional parameter *a* in Equation (3) may help dealing with heterogeneous substitution rates among residue pairs (e.g. 36). Hence, the relationship between GTR distances *d* and the corresponding *p*-distances *p* can be approximated by the EI transformation (3) with *a* = 4.590 ( Figure 3). Moreover, as *d* returned by Equation (3) is inversely proportional to *a* (for any fixed *p*), the relationship between *d* and *p* under the model GTR+Γ (with Γ shape parameter of 0.314, on average) can also be approximated by the EI transformation (3) with *a* = 0.291 ( Figure 3). 

These results show that complex distance measures can be approximated by simple analytical formulae based on few parameters. In practice, nucleotide frequencies (four parameters) can be trivially computed and *p*-distances (a fifth parameter) can be estimated using MH tools (see above). Therefore, the evolutionary distance *d* between two sequences that have evolved under the parameter-rich model GTR+Γ can be approximated from these only five parameters using (3) with *a* ≤ 4.590 ( Figure 3).

**Figure 4.**
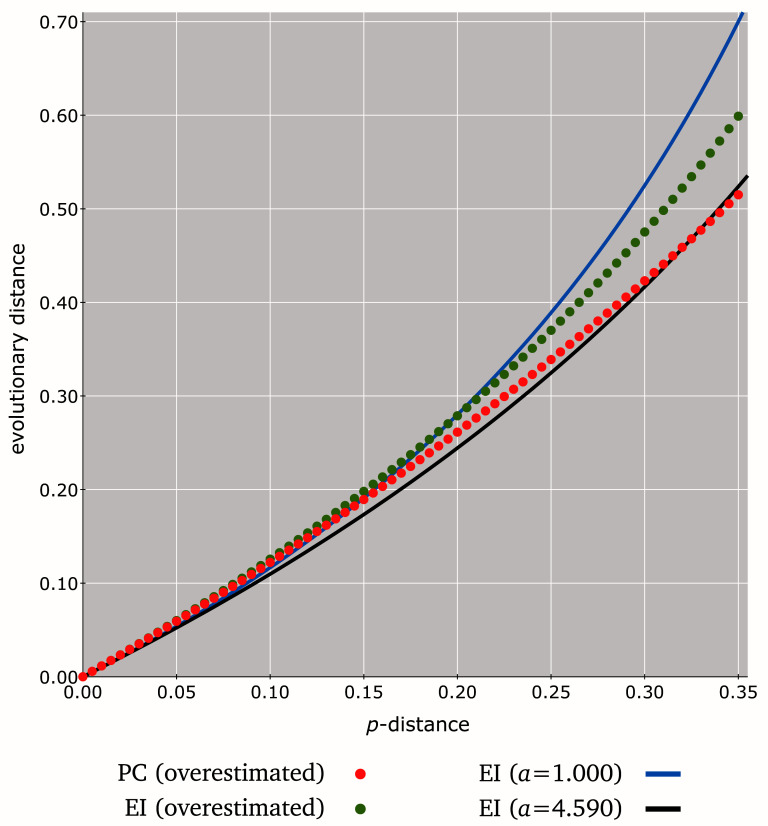
Impact of the MH *p*-distance upward bias on PC and EI transformations. The relationship between the *p*-distance *p* ( *x*-axis ranging from 0.00 to 0.35) and the corresponding evolutionary distance *d* ( *y*-axis ranging from 0.00 to 0.70) is represented when using PC (red dots) and EI (with equilibrium frequencies *f*_2_; green dots) transformations (2) on $\hat{p}$ = *β p* with *β* = 1.15. For ease of comparison with Figure 3, EI ( *f*_2_) transformation (3) on *p* are represented with *a* = 1.000 (blue curve) and *a* = 4.590 (black curve).

At this point, it should be stressed that MH $\hat{p}$ tends to be overestimated. Indeed, MH estimates are of the form $\hat{p}$ ≈ *βp* with slope *β* varying from 1.08 (BinDash, *k* = 31, *s* = 0.2) to 1.15 (Dashing, *k* = 26, *s* = 0.2) when *p* ≤ *p _r>_*_0.99 _( Figure 1). This has a direct impact on the derived distances: using PC and EI transformations (2) on $\hat{p}$ = *βp* with *β* = 1.15 and *p* ≤ 0.35 provide distance estimates that are quite comparable to the ones returned by Equation (3) with *a* ranging from 1.000 to 4.590 (see Figure 4 for the equilibrium frequencies *f*_2_; similar results were observed with *f*_1 _and *f*_3 _– not shown). The PC transformation (2) on the upward biased MH $\hat{p}$ returns distances that are then comparable to some complex distance measures (e.g. derived from a GTR model), therefore justifying its use by many MH tools. Nevertheless, the EI transformation (3) remains necessary when dealing with distantly related sequences (e.g. *p >* 0.2) and strong heterogeneity of the substitution rate across sites (e.g. often observed Γ shape parameter < 1.000). In such cases, the value of the parameter *a* should always be slightly increased to compensate the MH upward bias. For instance, EI transformation (3) on *p* with *a* = 0.291 (i.e. GTR+Γ distance least-square fitting in Figure 3) can be approximated by the same Equation on $\hat{p}$ = *βp* with *β* = 1.15 and *a* = 0.431.

### Phylogenetic reconstruction from MinHash-based evolutionary distances

To assess whether MH *p*-distance transformations may translate into reliable phylogenetic trees, additional simulations were performed. A total of 142 sets of sequences was simulated under the model GTR+Γ along reference phylogenetic trees. Representative GTR+Γ model parameters (same as above) and reference phylogenetic trees were obtained based on a ML analysis of real-case phylogenomics datasets. Sizes of the reference trees ranged from 10 to 154 taxa (31 on average), with diameters (i.e. maximum distance between any two leaves of a tree) varying from 0.204 to 2.883 (0.975 on average). Sequence lengths and indel events were simulated in the same way as the previous sequence pair simulations. 

**Figure 5.**
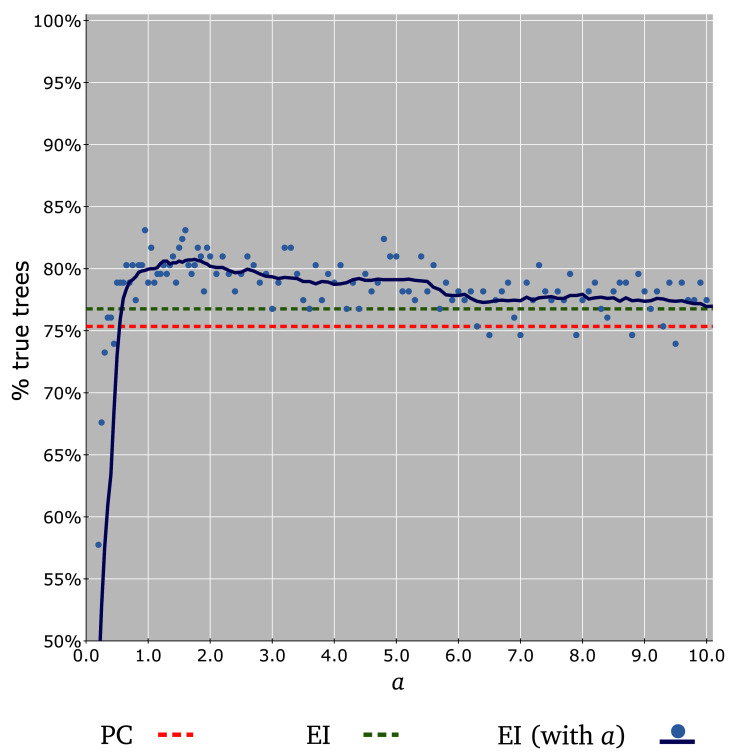
Accuracy of different *p*-distance transformations for phylogenetic inference. The percentage of recovered reference trees ( *y*-axis ranging from 50% to 100%) is represented (light blue dots) in function of the parameter *a* ( *x*-axis ranging from 0.0 to 10.0) in EI formula (3). The overall trend of these dots is illustrated using a moving average (dark blue curve). Dashed lines represent the percentages of recovered reference trees obtained with the PC (red) and EI (green) transformations (2).

The script JolyTree v2.0 was used to reconstruct phylogenetic trees from the simulated sequences. For each pair of unaligned sequences, this script estimates the MH *p*-distance using Mash, and transforms it into an evolutionary distance. Using these MH-based distances, JolyTree next reconstructs a minimum evolution phylogenetic tree with confidence supports at branches, based on a ratchet-based hill-climbing procedure (for more details, see 29). To obtain accurate MH *p*-distance estimates, JolyTree was run with parameters *q* = 10 ^−9 ^and *s* = 0.5 (see above). Evolutionary distances were estimated using the PC and EI transformations (2), as well as the EI transformation (3). To observe the impact of the parameter *a*, the EI transformation (3) was computed with *a* varying from 0.05 to 10.0. The accuracy of each *p*-distance transformation for phylogenetic inference was assessed by the percentage of recovered reference trees, i.e. identical topologies ( Figure 5).

Using JolyTree with EI transformations improves the percentage of recovered reference trees ( Figure 5). In spite of their limitations, PC distances result in the recovery of 75.3% of the 142 reference trees, but EI transformation (2) increases this percentage to 76.7% ( Figure 5). Furthermore, the EI transformation (3) generally provides better results in a large range of *a*, i.e. up to 83.1% of recovered reference trees ( Figure 5). Low *a*-values (e.g. *a* ≤ 0.3) translate into many incorrect tree topologies, whereas high ones (e.g. *a >* 6) tend to provide the same reference tree recovering percentage as the EI transformation (2) ( Figure 5). Most suitable values of *a* (corresponding to the highest reference tree recovering percentages, e.g. 80%) seem to range in the interval [1.0, 2.0] ( Figure 5).

These simulation results are consistent with two views which are somehow contradictory. On the one hand, accurate (parameter-rich) distance estimates are required, because biased ones (i.e. corresponding to a concave or convex function of the actual evolutionary distances) may result in incorrect phylogenetic trees ^23, 37^. On the other hand, simple (underparameterized) distance estimates should often be preferred, because they frequently result in more accurate tree topologies ^21, 38– 42^. Here, the simple PC and EI transformations (2) (one and five parameters, respectively) enable many reference trees to be recovered ( Figure 5). However, the EI transformation (3) is able to approximate realistic distance measures (e.g. GTR+Γ) by using only one supplementary parameter *a* ( Figure 3). It therefore enables more reference trees to be recovered ( Figure 5). 

In line with 43, most suitable values of *a* (e.g. between 1.0 and 2.0) are all higher than the Γ shape parameter values used for simulating the sequence datasets (i.e. varying from 0.162 to 0.422, with an average of 0.314). This can be explained by the MH upward bias (see above), but also by the large variance of the estimate (3) when *a* becomes low. Reminding that the Γ shape parameters (and the reference trees) used in these simulations were inferred from real-case datasets, these results suggest that using EI transformation (3) with *a* ≈ 1.5 may be suitable to infer genus phylogenetic trees. In light of this, it should be stressed that the article ^29^ describing the first distributed version of JolyTree (v1.1) incorrectly stated that the Mash output is the MH estimate $\hat{p}$ (instead of its PC transformation). As JolyTree v1.1 uses the EI transformation (2), this misinterpretation translates into the odd transformation formula *δ* = − *b*_1 _log *_e_* (1 + log _*e*_(1 − $\hat{p}$) */ b*_2_). However, as *δ* can be approximated on $\hat{p}$ ≤ 0.35 by the EI transformation (3) with *a* = 1.208, this explains the overall accuracy of JolyTree v1.1 despite its use of *δ* ^29^.

## Conclusions

Alignment-free phylogenetic inference from pairwise MH-based distance estimates is a promising approach. It enables phylogenetic trees to be quickly reconstructed from a large number of genomes without the burden of multiple sequence alignments (see e.g. 2, 29, 44– 52). This report confirms this view by showing that proper evolutionary distances can be easily derived from MH *p*-distance estimates, therefore enabling accurate phylogenetic inferences. 

First, although implemented to approximate nearest neighbors in sequence sets, current MH tools (e.g. Mash, BinDash, Dashing) were shown to be able to conveniently estimate pairwise *p*-distances *p* up to *p* ≈ 0.35. In practice, as *p* is very similar to the one-complement of the Average Nucleotide Identity (ANI; e.g. 29, 53, 54), MH estimates of *p* can then be obtained between genomes gathered from many bacteria, archaea or eukaryota genera, i.e. with pairwise ANI *>* 65%.

Second, the EI *p*-distance transformation (3) was proven efficient to approximate complex distance measures, e.g. derived from GTR model with heterogeneous substitution rates across sites. Because of an upward bias observed in MH *p*-distance estimates, simpler transformations (based on few parameters, as the commonly used PC) still provide distance measures that are comparable to GTR ones, but with (unrealistic) homogeneous substitution rates across sites. However, thanks to its supplementary parameter *a*, EI transformation (3) remains necessary to approximate distance measures between distantly related sequences that have arisen from more realistic substitution events. 

Third, as proper evolutionary distances can be derived from MH *p*-distance estimates, their efficiency in phylogenetic inference was established using the dedicated tool JolyTree ^29^. In particular, the EI transformation (3) with *a* ≈ 1.5 enables accurate phylogenetic trees to be inferred.

## Methods

### Model parameter estimation

To simulate the evolution of nucleotide sequences according to realistic substitution processes, the 187 genus datasets compiled in 29 (available at https://doi.org/10.3897/rio.5.e36178.suppl2) were first considered to infer a representative range of GTR parameter values. For each of the 187 genera, the associated genome assemblies were processed using Gklust v0.1 to obtain one representative genome assembly for each putative species. This analysis provided 142 sets of representative genome assemblies after discarding genera containing *<* 10 putative species. For each of these 142 sets, coding sequences were clustered using Roary v3.12 ^55^. Each cluster with at least four coding sequences was used to build a multiple amino acid sequence alignment using MAFFT v7.407 ^56^. Multiple sequence alignments were back-translated at the codon level and concatenated, leading to 142 supermatrices of nucleotide characters. A phylogenetic tree was inferred from each supermatrix of characters using IQ-TREE v1.6.7.2 ^57^ with evolutionary model GTR+Γ. All data related to these analyses are publicly available as *Extended data* at https://doi.org/10.5281/zenodo.4034244 ^58^.

### Sequence simulation

To assess the accuracy of different pairwise distance estimates, a simulation of sequence pairs was performed under both models GTR and GTR+Γ with three different sets ( *π*_A_, *π*_C_, *π*_G_, *π*_T_) of equilibrium frequencies: *f*_1 _= (0.25, 0.25, 0.25, 0.25), *f*_2 _= (0.10, 0.30, 0.40, 0.20), and *f*_3 _= (0.40, 0.10, 0.10, 0.40). For each of these six scenaria and for each *d* varying from 0.05 to 1.00 (step = 0.05), the program INDELible v1.03 ^59^ was used to simulate the evolution of 200 sequence pairs with *d* substitution events per character. Initial sequence length was 5 Mbs, and an indel rate of 0.01 was set with indel length drawn from [1, 50000] according to a Zipf distribution with parameter 1.5. For each simulated sequence pair, model parameters (i.e. GTR: six relative rates of nucleotide substitution; GTR+Γ: six rates and one Γ shape parameter) were randomly drawn from the 142 sets of estimated ones (see above). All simulated sequences are publicly available as *Extended data* at https://doi.org/10.5281/zenodo.4034461 ^60^. 

To compare the efficiency of *p*-distance transformations for phylogenetic reconstruction, the program INDELible v1.03 was also used to simulate the evolution of a sequence along each of the 142 phylogenetic trees previously inferred from different genera (see above). For each of the 142 genera, sequence evolution was simulated under the model GTR+Γ with the corresponding parameters (i.e. four nucleotide frequencies, six relative rates, and one Γ shape parameter). Sequence length and indel events were simulated as described above. The 142 simulated sequence sets are publicly available as *Extended data* at https://doi.org/10.5281/zenodo.4034643 ^61^.

### Sequence and phylogenetic analyses

MH *p*-distances were estimated with Mash v2.2, BinDash v1.0, and Dashing v0.3.4-11-gb44a. BinDash and Dashing were used with the MH *b*-bit ﬂavor with *b* = 18. Of note, as Mash and Bindash directly return the PC distance *d*, the corresponding *p*-distance was computed by *p* = 1 − *e* ^− *d*^. 

Phylogenetic tree reconstructions from simulated sequences were performed with the script JolyTree v2.0. This version implements the PC and EI transformations (2) and (3) of the pairwise *p*-distances estimated by Mash. If any, missing evolutionary distances *d _uv_* = ∅ (i.e. corresponding to $\hat{j}$ = 0 or *p* ≥ *b*_2_) between sequences *u* and *v* are approximated by JolyTree from the other non-missing evolutionary distances by $d_{uv}=\min_{x\neq u,v;\;d_{xu},d_{xv}\neq \emptyset }(d_{xu}+d_{xv})$. This fast approximation is derived from the triangle inequality property *d _uv_* ≤ *d _xu_* + *d _xv_* expected from the triplet of evolutionary distances induced by any sequence triplet *u*, *v*, *x* (see e.g. 62).

## Data availability

### Source data

A list of the 14,244 genome assemblies used to build the 187 genus datasets (Supplementary material of 29). https://doi.org/10.3897/rio.5.e36178.suppl2. 

### Extended data

Zenodo: Phylogenomic analyses of 142 prokaryotic genera. https://doi.org/10.5281/zenodo.4034244 ^58^.

Zenodo: Simulated pairs of nucleotide sequences for testing (alignment-free) genome distance estimate methods. https://doi.org/10.5281/zenodo.4034461 ^60^.

Zenodo: Model trees and associated simulated nucleotide sequences for testing phylogenetic inference methods. https://doi.org/10.5281/zenodo.4034643 ^61^.

Data are available under the terms of the Creative Commons Attribution 4.0 International license  (CC-BY 4.0). 
