# Supplementary material for: On the transformation of MinHash-based uncorrected distances into proper evolutionary distances for phylogenetic inference
Source: F1000Res. 2020 Nov 10;9:1309. [Version 1] doi: 10.12688/f1000research.26930.1 (PMC7713896; doi:10.12688/f1000research.26930.1)
Supplement: Peer review history

# Peer review: Reviewer response for version 1

**DOI:** 10.5256/f1000research.29746.r74631

**Published:** 2020-12-03

**Authors:** Guy Perrière

**Affiliations:** 1 Laboratoire de Biométrie et Biologie Evolutive, CNRS, UMR5558, Université Claude Bernard Lyon 1, Villeurbanne, France

I have no special criticism on the methodology and my comments on that part are really minor. First, what is the justification to the choice of a value equal to 1.5 for the shape of the Zipf distribution used to simulate the indels. As a user of INDELible myself I know very well how this value can be of importance so I would like to know how this value was chosen (arbitrary choice?). Second, I am not sure that the right reference for the computation of missing distances from the triangle inequality property is Guénoche and Grancolas (1999). The first paper I have seen on that topic is Lapointe and Kirsch (1995 ^1^). Also, I have no concerns on the results presented and everything seems ok for me.

 The conclusion of the manuscript presents the approach of phylogenetic inference from pairwise MH-based distance estimates as a possible alternative to classical methods of phylogenetic reconstruction (especially in bacteria). Indeed, a classical phylogenomic approach involves the chaining of a complex set of procedures: the identification of orthologous genes in multiple species; the alignment of these genes; and finally, the building of a tree from the concatenation of those alignments. I think that the inclusion in the paper of a comparison of two phylogenies obtained with: i) the MH-based distance and ii) a concatenation would strengthen this claim. A lot of concatenation datasets extracted from complete bacterial genomes are available and it would be not too difficult to do the experiment. I think this addition is of importance because I have no idea on the influence horizontal gene transfers can have on MH-based reconstructions when classical approaches try to minimize this influence.

Is the work clearly and accurately presented and does it cite the current literature?

Yes

If applicable, is the statistical analysis and its interpretation appropriate?

Yes

Are all the source data underlying the results available to ensure full reproducibility?

Yes

Is the study design appropriate and is the work technically sound?

Yes

Are the conclusions drawn adequately supported by the results?

Yes

Are sufficient details of methods and analysis provided to allow replication by others?

Yes

Reviewer Expertise:

Phylogenetics, comparative genomics and bioinformatics

I confirm that I have read this submission and believe that I have an appropriate level of expertise to confirm that it is of an acceptable scientific standard.

## References

[1] : Estimating Phylogenies from Lacunose Distance Matrices, with Special Reference to DNA Hybridization Data. *Molecular Biology and Evolution*.1995;12(2) : 10.1093/oxfordjournals.molbev.a040209 266-284 10.1093/oxfordjournals.molbev.a040209 Reference source

# Peer review: Reviewer response for version 1

**DOI:** 10.5256/f1000research.29746.r74629

**Published:** 2020-12-01

**Authors:** Burkhard Morgenstern

**Affiliations:** 1 Department of Bioinformatics, Göttingen Center for Molecular Biosciences, Institute for Microbiology and Genetics, University of Göttingen, Göttingen, Germany

The author investigates how phylogenetic distances between genomic sequences, calculated by MinHash methods, can be transformed into more meaningful distances, based on complex models of nucleotide substitutions.

 In the last few years, a number of alignment-free methods have been developed to calculate distances between genomic sequences. While earlier alignment-free methods used rough measures of sequence (dis)similarity, some methods have been proposed recently that accurately estimate distances - the number of substitutions per position since two sequences evolved from their last common ancestor - based on stochastic models of evolution (see below). These methods are restricted, however, to the simplest possible model, the Jukes-Cantor model.

 In recent papers, MinHash techniques were proposed as an attractive way of estimating the p-distance between DNA sequences, i.e. the number of mismatches per position in an (unknown) alignment of the compared sequences.

 The present paper shows how distances based on complex models of evolution (GTR, GTR + \Gamma) can be approximated using MinHash-based p-distances. The approach is carefully evaluated based on simulated sequences.

 The proposed method is a welcome and useful addition to existing alignment-free methods, extending them to more realistic models of evolution. The approach is novel, the paper is very well written and clear, and suitable references are given to the literature for more details. Therefore, I support indexing of the manuscript.  

 A certain limitation is that the manuscript is restricted to MinHash distances. According to the author, these methods are accurate for p-distances roughly < 0.25 (for reasonable k-mer length), but are less accurate for larger distances. However, a number of other alignment-free methods have been proposed that accurately estimate distances for much larger distances, e.g. Kr (Haubold *et al.*, 2009 ^1^), FSWM (Leimeister *et al.*, 2017 ^2^), Phylonium (Klötzl and Haubold, 2019 ^3^), Slope-SpaM (Röhling *et al.*, 2020 ^4^). All these methods estimate distances based on the simple Jukes-Cantor model; the program Co-phylog estimates non-corrected p-distances (Yi and Jin, 2013 ^5^). It should be straight-forward to transform these distances to more complex models, as done in the present paper, and to compare them to the MinHash methods evaluated in the paper.

Is the work clearly and accurately presented and does it cite the current literature?

Yes

If applicable, is the statistical analysis and its interpretation appropriate?

Not applicable

Are all the source data underlying the results available to ensure full reproducibility?

Yes

Is the study design appropriate and is the work technically sound?

Yes

Are the conclusions drawn adequately supported by the results?

Yes

Are sufficient details of methods and analysis provided to allow replication by others?

Yes

Reviewer Expertise:

Software development for sequence comparison and phylogeny. Alignment-free sequence comparison.

I confirm that I have read this submission and believe that I have an appropriate level of expertise to confirm that it is of an acceptable scientific standard.

## References

[1] : Estimating mutation distances from unaligned genomes. *J Comput Biol*.2009;16(10) : 10.1089/cmb.2009.0106 1487-500 10.1089/cmb.2009.0106 19803738

[2] : Fast and accurate phylogeny reconstruction using filtered spaced-word matches. *Bioinformatics*.2017; 10.1093/bioinformatics/btw776 10.1093/bioinformatics/btw776 28073754PMC5409309

[3] : Phylonium: fast estimation of evolutionary distances from large samples of similar genomes. *Bioinformatics*.2020;36(7) : 10.1093/bioinformatics/btz903 2040-2046 10.1093/bioinformatics/btz903 31790149PMC7141870

[4] : The number of k-mer matches between two DNA sequences as a function of k and applications to estimate phylogenetic distances. *PLoS One*.2020;15(2) : 10.1371/journal.pone.0228070 e0228070 10.1371/journal.pone.0228070 32040534PMC7010260

[5] : Co-phylog: an assembly-free phylogenomic approach for closely related organisms. *Nucleic Acids Res*.2013;41(7) : 10.1093/nar/gkt003 e75 10.1093/nar/gkt003 23335788PMC3627563

# Peer review: Reviewer response for version 1

**DOI:** 10.5256/f1000research.29746.r74633

**Published:** 2020-11-26

**Authors:** Brian Ondov

**Affiliations:** 1 Genome Informatics section, National Human Genome Research Institute, USA, Bethesda, MD, USA

The manuscript describes a series of simulations assessing various parametric transformations of MinHash-based genomic distance estimates into more robust evolutionary distances. It concludes that (1) MinHash methods accurately estimate nucleotide distances, (2) that accurate estimates of corrected evolutionary distances can be derived from these, with the more parameterized of two models performing better, and (3) that accurate phylogenies can be inferred from those estimates.

 The manuscript is well written and generally clear, although some of the methods are described so technically as to be hard to follow (e.g. p _max_ and p _r>0.99_ in "MinHash-based p-distance approximation").

 My main concern, which is not large, is the choice of the sketch size, *s *(or σ). The MinHash family of algorithms assumes *s* << *G* (genome size). The smallest sketch size tested here, however, is 1/5 of the average genome size. This size, described as "moderately large," is actually two or three orders of magnitude larger than Mash's default, for example. The justification for this is to lower the error bounds. As a consequence, however, what is being tested here is, in a sense, the viability of pure k-mer counting a la Fan *et al.*, rather than the MinHash approaches, which trade some accuracy of the Jaccard estimation for speed. This is still useful, but could be somewhat misleading for those that need the speed benefits of running MinHash with more typical parameters. Additionally, since the sweep of *s* is linear, but MinHash error bounds relate to an exponential of *s*, the sweep ends up being less informative than it could be, which is likely why the columns of Figure 1 are nearly identical.

 As a simple remedy, I would suggest sweeping the parameter *s* exponentially rather than linearly, starting closer to the default sketch size for the tools. It could still end closer to the genome size to get a sense of the behavior of MinHash as it degenerates to the actual k-mer Jaccard score. Along these lines, it would also make sense for the later phylogenetic experiments to either use a smaller *s* or do another sweep of values.

 As a minor point when listing relevant software in the introduction: Dashing is based on HyperLogLog sketching, which is similar in concept to MinHash but algorithmically distinct.

Is the work clearly and accurately presented and does it cite the current literature?

Yes

If applicable, is the statistical analysis and its interpretation appropriate?

Yes

Are all the source data underlying the results available to ensure full reproducibility?

Yes

Is the study design appropriate and is the work technically sound?

Yes

Are the conclusions drawn adequately supported by the results?

Yes

Are sufficient details of methods and analysis provided to allow replication by others?

Yes

Reviewer Expertise:

High-Performance Computing, Locality Sensitive Hashing, Sequence Alignment

I confirm that I have read this submission and believe that I have an appropriate level of expertise to confirm that it is of an acceptable scientific standard.
